# Synucleinopathies Take Their Toll: Are TLRs a Way to Go?

**DOI:** 10.3390/cells12091231

**Published:** 2023-04-24

**Authors:** Gabriella M. Mazzotta, Nadia Ceccato, Carmela Conte

**Affiliations:** 1 Department of Biology, University of Padova, 35131 Padova, Italy; 2 Department of Pharmaceutical Sciences, University of Perugia, 06100 Perugia, Italy

**Keywords:** Alpha-synucleinopathies, Toll-like receptors, non-motor symptoms

## Abstract

The misfolding and subsequent abnormal accumulation and aggregation of α-Synuclein (αSyn) as insoluble fibrils in Lewy bodies and Lewy neurites is the pathological hallmark of Parkinson’s disease (PD) and several neurodegenerative disorders. A combination of environmental and genetic factors is linked to αSyn misfolding, among which neuroinflammation is recognized to play an important role. Indeed, a number of studies indicate that a Toll-like receptor (TLR)-mediated neuroinflammation might lead to a dopaminergic neural loss, suggesting that TLRs could participate in the pathogenesis of PD as promoters of immune/neuroinflammatory responses. Here we will summarize our current understanding on the mechanisms of αSyn aggregation and misfolding, focusing on the contribution of TLRs to the progression of α-synucleinopathies and speculating on their link with the non-motor disturbances associated with aging and neurodegenerative disorders.

## 1. Introduction

The term α-synucleinopathies defines a group of neurodegenerative disorders associated with pathological accumulation of α-Synuclein (αSyn) aggregates in neurons and non-neuronal cells including microglia, pericytes, astrocytes, and oligodendrocytes [1]. Clinically, α-synucleinopathies comprise Lewy body disease (LBD) and multiple system atrophy (MSA). LBD is associated with abnormal accumulation of insoluble aggregated αSyn in Lewy bodies (LBs) and Lewy neurites (LNs) of neurons [2] such as in Parkinson’s disease (PD), PD dementia (PDD), dementia with Lewy bodies (DLB), and other neurodegenerative disorders [3]. MSA is a different synucleinopathy which includes two major clinicopathological subtypes characterized by the presence of argyrophilic glial cytoplasmic inclusions (GCI) and neuronal loss accompanied by gliosis in the basal ganglia, cerebellum, pons, inferior olivary nuclei, and spinal cord [4]. GCI have been reported also to consist of αSyn; therefore, MSA and LBD are considered the two major subtypes of synucleinopathies [5].

Although most synucleinopathies are sporadic, mutations in the gene of human αSyn have been described in both familial and sporadic forms of these diseases. For example, familial forms of PD are caused by gene duplication, triplication, and multiplications or by point-mutations such A30P, E46K, H50Q, G51D, A53T, and A53E [6,7,8].

αSyn is a small protein composed by 140 amino acids belonging to the synucleins family (α-synuclein, β-synuclein, γ-synuclein, and synoretin), whose members share high sequence identity and expression pattern [9]. Synucleins are natively unfolded proteins characterized by an acidic carboxyl terminus and an amino terminus containing imperfect repeat motifs (KTKEGV) [10]. Human αSyn is predominantly expressed in the brain and highly abundant in the presynaptic terminal of dopaminergic neurons. Phosphorylation and dephosphorylation of residue of serine 129 (pS129-αSyn) are known to be responsible for promoting or inhibiting the α-Syn aggregation, respectively [11,12,13]. However, the pathogenic relevance of pS129-αSyn remains controversial, as a recent finding shows that pS129-αSyn inhibits fibril formation and seeded aggregation [12]. It has been reported that the truncation of C-terminus of recombinant αSyn promotes its assembly to potentially form pathological filaments [14,15].

Although implicated in numerous cellular processes, the exact function of αSyn is still unclear. Under physiological conditions, it is soluble and may be involved in the compartmentalization, storage, and recycling of neurotransmitters, while pathological conditions can induce abnormal fold and formation of β-sheets, generate toxic and aggregated oligomers, and/or polymerize to produce intracellular plaques responsible for clinical signs [16]. In addition, nitration or cleavage are both associated with a greater tendency to aggregate [17,18,19]. 

Predominantly cytosolic and partly nuclear, αSyn can also be localized at the level of lipid membranes. The presence of αSyn was also found at the level of the mitochondrial membranes, internal and external, where it interferes with the activity of complex I and IV of the respiratory chain. Impairment of these complexes can lead to increased ROS production, often a cause of neuronal death [20,21,22]. αSyn is able to inhibit phospholipase D2, a membrane enzyme involved in the release of phosphatidic acid, useful for the formation of membranous vesicles and synaptic membranes [23,24,25] and to interact with different synaptic proteins by modulating their activity. In particular, its interaction with small GTPases (Rab) has been shown to be important in modulating membrane trafficking, exocytosis and synaptic vesicle release [26,27,28,29]. It is important to underline that the protein-protein interactions at the cytosolic level can influence the physiological and biochemical functions of αSyn and consequently its tendency to aggregate. This suggests that it is possible to modify the aggregation of αSyn by selectively regulating the expression of its protein partners [30,31].

Given its role in PD, PDD, LBD, and other synucleinopathies, in vitro and in vivo models can be valuable tools for studying the dopaminergic neuronal loss and the widespread of αSyn aggregates in different areas of the brain. 

Many patients with synucleinopathies experience motor symptoms that represent a hallmark of the disease. They include tremors (rhythmic shaking of a limb), rigidity (stiffness and resistance to movement), bradykinesia (slowness of movement), postural instability (difficulty with balance and coordination, ataxia (unsteady gait and lack of coordination), myoclonus (sudden, brief involuntary muscle jerks), and dysarthria (difficulty with speech and articulation). These motor symptoms are the result of a reduction in dopamine levels in the striatum and appear gradually and worsen over time as the disease progresses [32]. The specific symptoms and their severity can vary depending on the type of synucleinopathy and the progression of the disease can vary widely between individuals. In PD, it is estimated that up to 80% of dopaminergic cells in the substantia nigra may be lost before the characteristic motor symptoms become apparent [33,34]. 

## 2. Protein Aggregation and Propagation in Synucleinopathies

The proteostasis network works to maintain the proper balance between protein synthesis, folding, and degradation. It includes the ubiquitin-proteasome system, autophagy, chaperons, and heat shock proteins. Together, these pathways are responsible for regulating the number and the quality of proteins in the cell as well as their clearance. 

Perturbations in proteostasis have been associated with the pathogenesis of synucleinopathies [6]. αSyn is an intrinsically disordered protein that does not have a single, well-defined three-dimensional structure, but exists in a dynamic equilibrium of multiple conformations. While the normal expression of αSyn has a role in protecting cells against oxidative stress and apoptosis, its accumulation and the consequent formation of particular states of aggregation, such as oligomers or fibrils, may be harmful to the cell. It has been demonstrated that in certain conditions, such as oxidative stress, nitrosative stress, and inflammation, αSyn can misfold into amyloid-like fibrils. Once misfolded, αSyn can form cell-to-cell aggregates which can spread throughout the brain and cause further damage [6,35]. This hypothesis has been further supported by other studies, which have found that αSyn expression levels can influence the progression of diseases such as PD [36], Alzheimer disease (AD) [37], and Huntington’s disease (HD) [38]. Although it is still debated whether αSyn aggregation is the main leading cause for the neuronal death, to date, oligomeric/aggregated species of αSyn contained in the LBs and LNs are considered the hallmarks of PD and other synucleinopathies [39,40,41]. There is a different susceptibility of specific brain areas to LB and LN formation. For example, due to the presence of numerous neurites and terminations and the high requirement for mitochondrial activity, dopaminergic neurons represent the cells most vulnerable to LB and LN build-up [42,43,44]; LBs and LNs are preferentially formed in nigrostriatal neurons with long-range projections [45,46,47]. Hyperbranching axons as in the nigrostriatal system may facilitate αSyn deposition [48]. These neurons are more vulnerable to oxidative and nitrosative stress [49,50,51,52]. It has been proposed that the progression of Lewy pathology is from axon terminals to neuronal soma. However, a “mitocentric view of PD” claims that mitochondrial dysfunctions can lead to nigrostriatal degeneration regardless of Lewy pathology [48]. 

The exact mechanism of αSyn misfolding is still not fully understood, even if it is thought to involve a combination of environmental and genetic factors [53,54,55]. Studies have suggested that misfolding may be caused by exposure to toxins, age-related alterations in the αSyn structure, and gene mutations, and may cause an increased propensity to the formations of αSyn aggregates and fibrils [42,55]. 

αSyn aggregation initiates by a short sequence of a seed or native, partially folded or unfolded oligomers, which adopt a non-native conformation and auto-assemble into higher-order oligomers. These oligomers can serve as precursors of fibril nucleus, highly dynamic species that recruit other monomers eliciting a rapid polymerization into amyloid fibrils, hierarchical polymorphic stable structures derived from protofibrils that are responsible for the development of several diseases. The current goal is to identify the exact intermediate structures and pathways involved in certain diseases and that are characteristic for each individual. Since amyloid fibril-associated diseases are orphan drug, building a molecular profile for a group of individuals with certain feature clinical signs could improve the diagnosis and be helpful for the designing of molecules able to potentially prevent the pathological escalation. 

It is believed that the pathological process of synucleinopathies begins in the anterior olfactory nucleus and dorsal motor nucleus of the glossopharyngeal and vagal nerves and then spreads to other regions of the brain, such as the basal ganglia, thalamus, and cortex following a stereotyped pattern [56,57]. It is also suggested that αSyn could spread from the gut to brain via the vagus nerve [58,59].

Cell-to-cell propagation of αSyn pathology is also a feature of PD and related synucleinopathies [60,61,62,63]. Studies have found that misfolded αSyn can spread between neuroanatomically-connected regions of the brain, from neuron to neighboring cells such as other neurons, and microglial and astroglial cells in a prion-like manner. Dissemination of αSyn produces toxic aggregates, triggering a neuroinflammatory status that contributes to symptoms worsening over time and ultimately to neuronal death [63,64].

The precise mechanisms driving αSyn propagation are not completely known, but are believed to involve a combination of exocytosis and endocytosis and several pathway and cellular responses, including the release of αSyn-containing exosomes responsible for activation of microglial cells [65]. The internalization of αSyn seeds can also take place via transmembrane, transsynaptic endocytosis, phagocytosis, receptor-mediated endocytosis, cell injury and leaking, and tunneling nanotubes, the special membranous bridges containing F-actin connecting the cytoplasm of neighbor cells [66]. It has been shown that exosomes extracted from plasma of PD patients contain monomeric and oligomeric forms of αSyn that are crucial for the communication between cells and that aggregates of αSyn can be detected in extracellular vesicles derived from CSF of PD patients [67]. Moreover, cell-to cell transmission can involve the binding of αSyn to lymphocyte activation gene 3 and amyloid precursor-like protein 1 receptors [68].

αSyn can activate microglia and other immune cells and promote the production of pro-inflammatory cytokines, suggesting that immune activation and αSyn accumulation may feed back into a positive loop, thus exacerbating the neurodegenerative process in PD and other synucleinopathies [69]. In the next section, we will summarize the current understanding of the involvement of Toll-like receptor (TLR)-mediated neuroinflammation in the αSyn pathogenesis.

## 3. Toll-like Receptors (TLRs) in α-Synuclein Aggregation

The Toll-like receptors (TLRs) represent a family (at least 10 members TLR1-TLR10) of transmembrane proteins expressed by immune and non-immune cells, including microglia, neurons, astrocytes, and oligodendrocytes involved in the activation of the innate immune system [70]. They are located on the surface of cells and recognize pathogen-associated molecular patterns (PAMPs) derived from bacteria, viruses, fungi, and other pathogens or can also be found in the endosome and cytoplasm where they detect and respond to viral nucleic acids [71,72]. TLRs are also able to recognize a wide variety of damage- or danger-associated molecular patterns (DAMPs), also known as alarmins, including αSyn, released by damaged neuronal cells and injured tissues [73]. Upon recognition of these molecules, TLRs activate signaling pathways that lead to the production of pro-inflammatory cytokines, and can recruit and activate other immune cells, such as T cells and B cells initiating an adaptive immune response [74,75,76]. 

Downstream signaling pathways of TLRs include the myeloid differentiation primary-response gene 88 (MyD88), MyD88-adaptor-like protein (MAL), TIR-domain-containing adaptor protein inducing interferon-β (IFNβ) (TRIF), TRIF-related adaptor molecule (TRAM), and sterile α- and armadillo-motif-containing protein (SARM). Except for TLR3, MyD88 is a key component of the TLR signaling pathway. It is a cytoplasmic adaptor protein that, upon recruitment, activates signaling molecules such as IL-1R-associated (IRAK) kinases, TNF receptor-associated factors (TRAFs), and TAK1 protein kinase complex, starting a signaling cascade that in turn leads to the activation of NF-kB and finally to the production of pro-inflammatory cytokines [77]. 

TLRs and αSyn are shown to be reciprocally influenced in a positive feedback loop; indeed, αSyn increases the expression of TLRs, including TLR1, TLR2, TLR3, and the adaptor Myd88 [77] and TLR2 and TLR4 are dysregulated in PD patients and animal models [78,79,80,81]. TLR dysregulation has been linked to the accumulation of misfolded α-syn and thereby widely implicated in the pathogenesis of the synucleinopathies [71,82]. However, there is still controversy in synucleinopathies regarding the advantageous or detrimental functions of TLRs, especially of TLR4. For example, the lack of TLR4 is associated with αSyn upregulation [83] and dopamine depletion [84]. Moreover, Stefanova et al. [85] showed that the ablation of LR4 prevented αSyn phagocytosis and clearance. However, another study using TLR4 knockout mice revealed less neuroinflammation and neurodegeneration [86]. Prolonged inflammation can promote αSyn misfolding but many other factors can contribute to the αSyn pathology. Synucleinopathies, including PD, arise from a combination of genetic and non-genetic factors. Processes ranging from neurons and glia interaction to protein-protein interaction and protein misfolding are known to cause neuronal death. Moreover, multimorbidities such as multiple proteinopathies and co-occurring vascular and metabolic dysfunctions together with aging, sex and genetic factors contribute to the neurodegeneration and influence the course of disease [87]. The presence of combinated proteinopathies and comorbidities has important implications for the research of novel biomarkers and for development of therapeutic targets and strategies [88].

Although most studies have focused on TLR2 and TLR4, this could also be true for other TLRs, including TLR7 and TLR9 [89]. In fact, TLR7 activation can lead to the accumulation of αSyn in the brain and TLR9 might also be involved in the activation of microglia and the production of pro-inflammatory cytokines in synucleinopathies [90]. Additionally, TLR7 inhibition has been shown to reduce the accumulation of αSyn in animal models of PD, suggesting that TLR7 may be a potential therapeutic target for the treatment of synucleinopathies [91]. In addition, TLR8 targeting by small molecule agents is proposed to have a potential clinical application [91,92].

αSyn activates leucine-rich-repeat and pyrin-domain-containing3 (NLRP3) inflammasome, generating extensive microgliosis [93]. In this scenario, TLRs attend both as “assembling signals” and as an “activation signal” for NLRP3 inflammasome activation responsible for pro-inflammatory cytokine release and thereby microgliosis and astrogliosis. It has been shown that αSyn aggregates released by injured neurons are recognized by TLR2 or TLR4 and can take divergent paths: they can be either moved to lysosome for degradation and clearance or promote the activation of NLRP3 inflammasome causing a diffuse αSyn proteotoxicity in several brain regions such as midbrain, hippocampus and cortex [94,95]. In the CNS, the presence of TLRs on the surface of “sentinel cells” such as microglia, neurons, and astrocytes, favors the intracellular uptake, transport, and degradation via lysosomal pathway. However, a partial degradation leads to the further intracellular accumulation and neuroinflammation [96]. 

**Figure 1:**
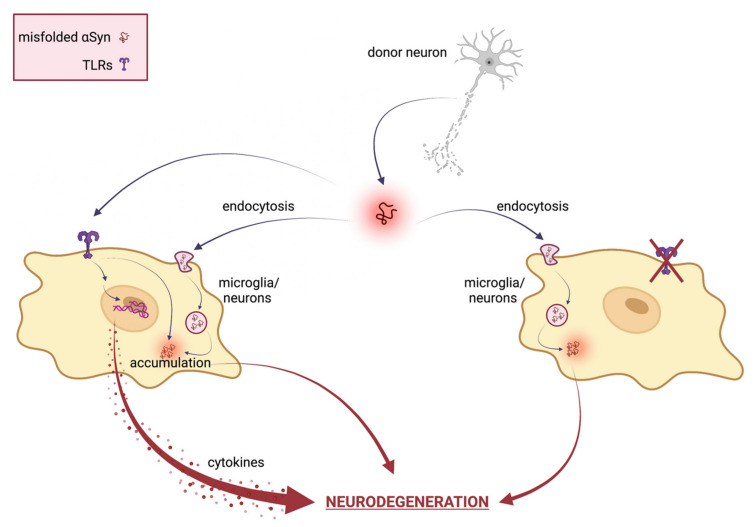
Schematical representation of TLRs involvement in αSyn mediated neurodegeneration. In neuronal cells, misfolded αSyn can be internalized via TLRs or several indirect endocytosis methods. Extracellular αSyn activates a TLR signaling cascade that results in neurotoxic responses, such as pro-inflammatory cytokine expression and release, ultimately leading to neuronal damage and pathological modification of αSyn. Therefore, TLR specific targeting ameliorates αSyn-mediated neurotoxicity by inhibiting TLR-mediated αSyn internalization and inflammatory response. Created with BioRender.com.

The αSyn proteostasis in the nervous central system is controlled by a selective autophagy pathway termed “synucleinphagy” that also requires the presence of TLRs. TLR-mediated activation of microglia could engulf αSyn into autophagosomes to be degraded; therefore, the disruption of synucleinphagy can cause the accumulation of misfolded αSyn and neuronal death. Various conformations of extracellular αSyn, including monomers, oligomers, and high-molecular-weight aggregates, can induce microglial neuroinflammation via TLRs [97]. In particular, both TLR2 and TLR4 can interact with αSyn, promoting its internalization into microglia [85,98,99]. Furthermore, targeting TLR2 has proven to be a good approach for inhibiting pathogenic cell-to-cell αSyn transmission and astroglial inflammatory responses, and for clearing toxic species via autophagic machinery. Once internalized, αSyn oligomers spread from neuron to glial cells, engaging and activating TLRs on nearby microglia surface and directly modulating its uptake and intracellular trafficking. Therefore, targeting TLRs and reactive microglia may be a promising therapeutic strategy also for preventing cell-to-cell transmission and slowing the progression of synucleinopathies. Figure 1 reports a schematic representation of the TLRs’ involvement in αSyn-mediated neurodegeneration.

The characterization of the novel pathogenic species of αSyn and unexplored mechanisms that underlie their aggregation and spreading represent major goals and TLRs represent good candidates to look at. Furthermore, since the activation of specific TLR members on the cell surface or in intracellular milieu depend on specific αSyn conformations [100,101,102], associating a single TLR with a specific altered species of synuclein could help expand knowledge on the mechanisms of the synucleinopathies and help the designing of TLR agonists.

In the plethora of animal models used to study PD and other neurodegenerative disorders, *Drosophila melanogaster* is taking the stage; indeed, the pathology observed in human PD can be accurately reproduced in Drosophila, with an area-specific and age-dependent loss of dopaminergic neurons, as well as LB and LN formation. Flies do not have the αSyn gene; however, the ectopic expression of human αSyn in the nervous system recapitulates cardinal features of Parkinson’s pathology [103], from the accumulation of protein aggregates to dopaminergic neurodegeneration, locomotor impairment, and lifespan reduction [104].

In Drosophila, the Toll family counts nine members (*Toll*, *18-Wheeler-,* and *Toll-3* to *Toll-9*), most of which are highly expressed during embryogenesis and metamorphosis and are involved in developmental regulation and in the onset of the immune response [105]. Drosophila TLRs do not function by recognizing different PAMP, as their mammalian counterparts do, and several of them exhibit also morphogenetic and neural functions [106]. For example, *Toll-6* and *Toll-7* are required for the development of the nervous system, either in proper targeting of motoneurons or in the development of the olfactory system [107]. *Toll-8* is involved in neuron-specific glycosylation and neural patterning and developmental neuromuscular junction (NMJ) growth [108]. The expression of Toll-related genes was analyzed in selected populations of cells across the brain: while *Toll-1*, *Toll-2*, *Toll-6*, *Toll-7* and *Toll-8* exhibited a differential expression in the various neuronal subpopulations, *Toll-4*, *Toll-5*, and *Toll-9* were not expressed by any of the CNS tested cells [109]. 

Evidence is increasing regarding the reciprocal relationship between Toll-mediated signaling pathway and neurodegeneration in Drosophila. In a fly model for AD disease, *Toll* loss-of-function mutations suppress the neuropathological effects of the Aβ42, while its overexpression or gain-of-function mutations enhance them [110]. Moreover, flies overexpressing the ALS-related TDP-43 ortholog (TBPH) exhibit increased expression levels of Toll pathway-related AMP genes and, at the same time, deletion of *Toll* or Toll-related genes reduces the TDP-43-related effect, thus improving lifespan and associated motility defects [111].

## 4. Non-Motor Symptoms in Synucleinopathies: A Focus on Circadian and Sleep Disturbances

The cardinal clinical feature of synucleinopathies, and neurodegenerative disorders in general, might be headed by a range of non-motor symptoms, such as cognitive impairment, autonomic dysfunction and circadian and sleep disturbances. These non-motor symptoms are increasingly relevant: on one hand they profoundly impact the patients’ quality of life and, on the other side, the fact that they precede motor symptoms and cognitive decline for many years suggests that in this pre-motor phase the pathogenic process is presumably underway and involves different regions of the peripheral and central nervous system [110].

Most human biological functions have specific temporal dynamics: biochemistry, physiology and behavior are temporally structured and characterized by a periodicity of about 24 h, in synchrony with the solar time. These rhythms are generated by an endogenous timekeeping system, the circadian clock, which regulates, among others, biological functions such as sleep/wake, feeding, body temperature, and hormonal levels. In mammals, the central pacemaker controlling behavioral and physiological rhythms is located in the suprachiasmatic nucleus (SCN), a bilateral structure in the anterior hypothalamus comprising about 20,000 clock neurons, each containing the molecular clock machinery [111]. At molecular level, endogenous oscillations are generated by interlocked transcriptional-translational feedback loops (TTFLs), in which positive elements promote the rhythmic transcription of the negative elements that, in turn, inhibit the activity of the positive elements [112]. 

One of the prominent circadian-related symptoms in neurodegenerative disease patients is the alteration of sleep/wake patterns; during disease progression, nighttime sleep becomes progressively more fragmented, with a consequent increase in nocturnal activity and daytime sleepiness [113]. These observations of poorly consolidated rest/activity patterns in humans are paralleled by animal models. Rodent models for PD display impairment in the sleep wake parameters, such as deficit in REM sleep, overwhelming episodes of sleep, like “sleep attacks”, and increased sleepiness [114,115,116]. Furthermore, the overexpression of αSyn in mice was reported to correlate to deficits in circadian locomotor activity [117].

Molecular and physiological rhythms are also altered in PD patients, in which the normal 24-h oscillation in the clock gene Bmal1 expression was lost and melatonin levels were reduced [118]. Several studies have reported diurnal fluctuation in symptoms and signs associated with PD, such as motor symptoms [118], visual performance [119], and responsiveness to dopaminergic treatments [118], leading to the hypothesis of a circadian influence on the expression of clinical features of PD. 

The hypothesis of an intimate connection between circadian sleep regulation and α-synucleinopathies is further reinforced by the fact that melatonin was shown to prevent formation of αSyn aggregation as revealed by immunostaining in a cell model for maneb-induced neurodegeneration [120] and by a combination of several in vivo and in vitro approaches that revealed the ability of melatonin to affect the αSyn conformational dynamics and assembly, and consequently, its cytotoxicity [121]. Furthermore, studies on animal models have suggested that the circadian disruption observed in PD is not just a secondary phenomenon but can rather be a contributing factor to its pathogenesis: indeed, in an MPTP (1-methyl-4-phenyl-1,2,3,6-tetrahydropyridine) mouse model of PD, which causes the selective destruction of dopaminergic neurons, animals that were subjected to chronic circadian disruption before receiving MPTP showed a worsening of their motor and cognitive deficits, as a result of an increased loss of dopaminergic neurons and a more intense neuroinflammatory response [122].

## 5. Circadian and Sleep Disorders in α-Synucleinopathies: TLRs Could Have a Say

Increasing evidence suggest that the circadian clock is closely associated with the immune system [123,124,125,126,127,128] and this association also involve TLRs [129]. Indeed, in vertebrates, the TLR9 gene expression is clock-controlled, with highest levels during the light phase and lower during the dark. This rhythmic expression is due to putative E-box sequences present in its promoter and ultimately results in a daily variation of the TLR9 mediated immune response [130].

In humans and animal models such as mouse and rat, the TLR4 ligand LPS can influence the expression of clock genes in the central pacemaker neurons, the suprachiasmatic nuclei, and peripherals; moreover, the levels of TLR4 were themselves reduced upon overexpression of the clock gene Cry1 [130,131].

TLR4 also mediates both the quality and the duration of sleep [131,132]; indeed, in light dark cycles, TLR4 KO mice exhibited an increased wakefulness in the early dark phase and a proportional reduction in the NREM sleep [131]. Both TLR2 and TLR4 contribute to sleep regulation: double TLR2/4 knock-out mice exhibit a 42% increase in REM sleep during daytime, and 41% more time awake during the night [132]. TLR2 is also involved in consolidating behavioral periodicity: young TLR2 KO mice show a reduced daily variability in rhythmic behaviors such movement, feeding, and drinking, in comparison to wild type [133]. 

**Figure 2:**
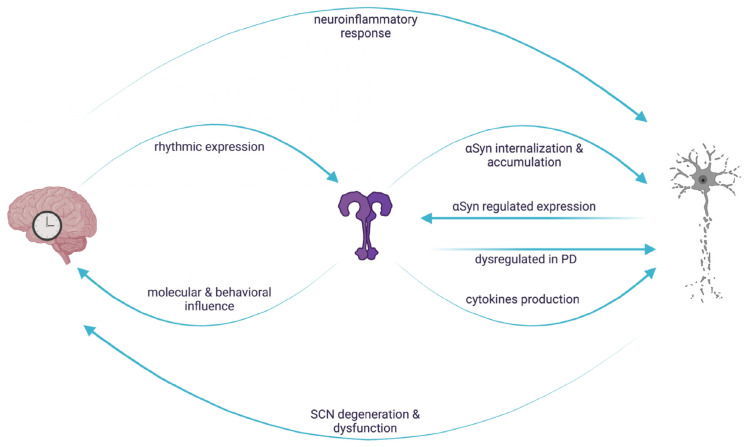
Reciprocal interaction between circadian regulation, TLR signaling and αSyn mediated neurodegeneration. In vertebrates, TLR expression is rhythmically controlled and in turn influences molecular and behavioral rhythms. Circadian clocks control the neuroinflammatory response leading to neuronal damage, which, in turn, causes circadian dysfunctions. The rhythmic regulation of TLRs could result in a daily variation of the downstream signaling cascades, conferring a circadian signature on αSyn-mediated neuronal degeneration. Created with BioRender.com.

A schematic representation of the reciprocal interaction involving TLRs, circadian machinery and neurodegeneration is reported in Figure 2.

## 6. Conclusions and Perspective

Although loss of neuronal cells represents the primary cause of the development and progression of synucleinopathies, the underlying mechanisms are intricate. αSyn misfolding promotes the formation of insoluble structures that are believed to contribute to the disruption of cellular processes, neuronal death, and development of symptoms. Astrocytes, microglia, oligodendrocytes, and neurons orchestrate the complex crosstalk between intra- and extracellular αSyn levels by activation of intra- and extracellular TLRs. However, the finding regarding the beneficial or detrimental role of TLRs are conflicting and the precise molecular mechanisms are not completely understood. Further research is needed to clarify the relationship between TLRs and α-synucleinopathies and to determine whether TLR-targeted therapies may hold promise for treating these diseases. Moreover, as circadian and sleep disorders precede the emergence of cognitive and motor symptoms by years, the possibility to identify individuals at early stages of neurodegeneration will be an important step towards earlier intervention aimed at slowing the progression of the disease and minimizing its clinical disturbances.

## Data Availability

Not applicable.
